# Genomic heterogeneity underlies multidrug resistance in *Pseudomonas aeruginosa*: A population-level analysis beyond susceptibility testing

**DOI:** 10.1371/journal.pone.0265129

**Published:** 2022-03-31

**Authors:** Laura J. Rojas, Mohamad Yasmin, Jacquelynn Benjamino, Steven M. Marshall, Kailynn J. DeRonde, Nikhil P. Krishnan, Federico Perez, Andrew A. Colin, Monica Cardenas, Octavio Martinez, Armando Pérez-Cardona, Daniel D. Rhoads, Michael R. Jacobs, John J. LiPuma, Michael W. Konstan, Alejandro J. Vila, Andrea Smania, Andrew R. Mack, Jacob G. Scott, Mark D. Adams, Lilian M. Abbo, Robert A. Bonomo

**Affiliations:** 1 Department of Molecular Biology and Microbiology, Case Western Reserve University School of Medicine, Cleveland, Ohio, United States of America; 2 Research Service, Louis Stokes Veterans Affairs Medical Center, Cleveland, Ohio, United States of America; 3 CWRU-Cleveland VAMC Center for Antimicrobial Resistance and Epidemiology (Case VA CARES), Cleveland, Ohio, United States of America; 4 The Jackson Laboratory for Genomic Medicine, Farmington, Connecticut, United States of America; 5 Jackson Memorial Hospital, Jackson Health System, Miami, Florida, United States of America; 6 Center for Proteomics and Bioinformatics, Case Western Reserve University School of Medicine, Cleveland, Ohio, United States of America; 7 Departments of Translational Hematology and Oncology Research and Radiation Oncology, Cleveland Clinic, Cleveland, Ohio, United States of America; 8 Medical Service, Louis Stokes Cleveland VA Medical Center, Cleveland, Ohio, United States of America; 9 CONICET, Centro de Investigaciones en Química Biológica de Córdoba (CIQUIBIC), Córdoba, Argentina; 10 Division of Infectious Diseases and HIV Medicine, Cleveland, Ohio, United States of America; 11 GRECC Louis Stokes Cleveland Veterans Affairs Medical Center, Cleveland, Ohio, United States of America; 12 Department of Pediatrics, University of Miami Miller School of Medicine, Miami, Florida, United States of America; 13 Division of Pulmonology, Department of Pathology University of Miami Miller School of Medicine, Miami, Florida, United States of America; 14 Department of Laboratory Medicine and Infection Biology Program, Cleveland Clinic, Cleveland, Ohio, United States of America; 15 Department of Pathology, Cleveland Clinic Lerner College of Medicine, Case Western Reserve University Cleveland, Ohio, United States of America; 16 Division of Pediatric Infectious Diseases, Department of Pediatrics, University of Michigan Medical School, Ann Arbor, Michigan, United States of America; 17 Department of Pediatrics, Case Western Reserve University School of Medicine and Rainbow Babies and Children’s Hospital, Cleveland, Ohio, United States of America; 18 Instituto de Biología Molecular y Celular de Rosario (IBR, CONICET-UNR), Rosario, Argentina; 19 Universidad Nacional de Córdoba, Facultad de Ciencias Químicas, Departamento de Química Biológica, Córdoba, Argentina; 20 Division of Infectious Diseases Department of Medicine University of Miami Miller School of Medicine, Miami, Florida, United States of America; 21 Department of Pharmacology, Cleveland, Ohio, United States of America; 22 Department of Biochemistry Case Western Reserve University School of Medicine, Cleveland, Ohio, United States of America; Suez Canal University, EGYPT

## Abstract

**Background:**

*Pseudomonas aeruginosa* is a persistent and difficult-to-treat pathogen in many patients, especially those with Cystic Fibrosis (CF). Herein, we describe a longitudinal analysis of a series of multidrug resistant (MDR) *P*. *aeruginosa* isolates recovered in a 17-month period, from a young female CF patient who underwent double lung transplantation. Our goal was to understand the genetic basis of the observed resistance phenotypes, establish the genomic population diversity, and define the nature of sequence evolution over time.

**Methods:**

Twenty-two sequential *P*. *aeruginosa* isolates were obtained within a 17-month period, before and after a double-lung transplant. At the end of the study period, antimicrobial susceptibility testing, whole genome sequencing (WGS), phylogenetic analyses and RNAseq were performed in order to understand the genetic basis of the observed resistance phenotypes, establish the genomic population diversity, and define the nature of sequence changes over time.

**Results:**

The majority of isolates were resistant to almost all tested antibiotics. A phylogenetic reconstruction revealed 3 major clades representing a genotypically and phenotypically heterogeneous population. The pattern of mutation accumulation and variation of gene expression suggested that a group of closely related strains was present in the patient prior to transplantation and continued to change throughout the course of treatment. A trend toward accumulation of mutations over time was observed. Different mutations in the DNA mismatch repair gene *mutL* consistent with a hypermutator phenotype were observed in two clades. RNAseq performed on 12 representative isolates revealed substantial differences in the expression of genes associated with antibiotic resistance and virulence traits.

**Conclusions:**

The overwhelming current practice in the clinical laboratories setting relies on obtaining a pure culture and reporting the antibiogram from a few isolated colonies to inform therapy decisions. Our analyses revealed significant underlying genomic heterogeneity and unpredictable evolutionary patterns that were independent of prior antibiotic treatment, highlighting the need for comprehensive sampling and population-level analysis when gathering microbiological data in the context of CF *P*. *aeruginosa* chronic infection. Our findings challenge the applicability of antimicrobial stewardship programs based on single-isolate resistance profiles for the selection of antibiotic regimens in chronic infections such as CF.

## Introduction

Multidrug resistant (MDR), extremely drug resistant (XDR) and pandrug resistant (PDR) infections are rapidly becoming the scourge of contemporary clinical medicine [1]. The World Health Organization and Centers for Disease Control and Prevention have both designated *Pseudomonas aeruginosa* as one of the major pathogens for which new antibiotics are desperately needed [2, 3]. Consequently, *P*. *aeruginosa* is a persistent and difficult-to-treat pathogen in many patients, especially those with cystic fibrosis (CF). CF is an inherited autosomal recessive multisystem disorder caused by mutations in the cystic fibrosis transmembrane conductance regulator gene (CFTR) on the long arm of chromosome 7. The absence of or dysfunction of the CFTR protein in the epithelial cell lining of ducts leads to excessive absorption, impaired muco-ciliary clearance, thickened secretions, and a consequent inability to clear bacteria, resulting in chronic airway inflammation [4]. *P*. *aeruginosa* possesses a versatile arsenal of antimicrobial resistance determinants and virulence factors that enable survival, adaptation, and consequent persistence within the complex milieu of CF. *P*. *aeruginosa* establishes early airway infection and cellular attachment via flagella and pili. Extended survival within the airways is further enabled via well described mucociliary clearance defects that promote additional binding and protection against phagocytosis [5, 6]. Several other mechanisms are known to contribute to the persistence of *P*. *aeruginosa* in the airways of CF patients. These include downregulation of toxin production [7], loss of lipopolysaccharide "O" side chains, biosynthesis of alginate coat polysaccharide, and loss of flagellum or pili to evade the host immune response [8]. According to the Cystic Fibrosis Foundation, the percentage of individuals with a positive culture for *P*. *aeruginosa* was 18.1% in 2020, of which 13.2% were reported to be MDR. Among CF patients, the rates of MDR-*P*. *aeruginosa* infection are highest in older adolescents and adults, likely reflecting prolonged exposure to antibiotics [9]. Strains that fulfil the more recently contrived definition of *P*. *aeruginosa* with difficult-to-treat resistance (DTR-*P*. *aeruginosa*), pose a particular threat to patients with CF [10, 11], as *P*. *aeruginosa* is the most prevalent pathogen in the lungs of adults with CF, and a major contributor to morbidity and mortality [10, 11]. Chronic *P*. *aeruginosa* infection is a major risk factor for loss of lung function, eventually causing respiratory failure and early death. At the point of respiratory failure, the only reprieve is lung transplantation, which brings forth the added risks of immunosuppression in the setting of chronic DTR-*P*. *aeruginosa* [12].

Prior extensive work has demonstrated an abundance of *P*. *aeruginosa* population and sub-population phenotype heterogeneity within CF patients since early childhood. Numerous physiologic properties unique to the lungs and bronchial airways of CF patients, contribute to the selection of *P*. *aeruginosa* variants with heightened mutation frequency [13]. Microbial and host factors furthermore impact the ongoing adaptive response exhibited by mutating strains within the airways of immunocompromised CF patients. This results in the acquisition of advantageous attributes such as virulence factor assembly, motility, antibiotic resistance, and metabolic adaptation [14]. Prior studies have shown that hypermutator strains may constitute up to 60% of all *P*. *aeruginosa* strains dwelling within the airways and lungs of CF patients. Whole genome sequencing (WGS) and RNAseq analysis has enabled the study of isolates collected sequentially from patients and the characterization of the evolutionary patterns and/or trajectories that comprise the hallmark complexity of diverse subclones of *P*. *aeruginosa* in CF patients.

Herein, we describe the population structure, genomic and phylogenetic complexity found in *P*. *aeruginosa* isolates collected longitudinally during a 17-month observation period (Nov 2016 –Mar 2018) in a young woman suffering from CF who underwent lung transplantation. Our aim was to establish the genomic population diversity and define the nature of genomic changes over time in the context of chronic CF infection.

## Materials and methods

### Clinical summary and selection of *P*. *aeruginosa* isolates

**Fig 1:**
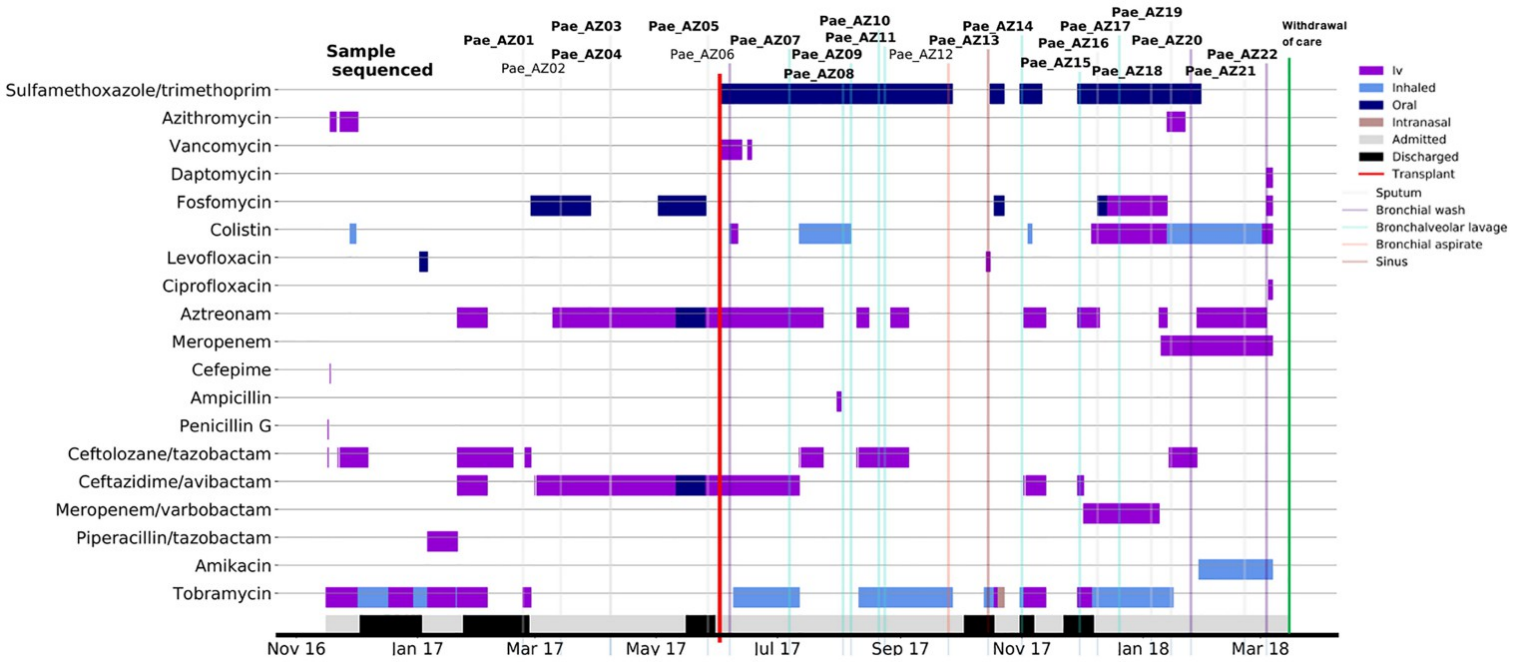
Timeline of antibiotic treatment and isolates recovered from the CF patient within the 17-month period until the withdrawal of care. Indicated in bold are isolates that underwent whole genome sequencing.

*P*. *aeruginosa* isolates included in this study were collected from sputum, bronchoalveolar lavage, or bronchial aspirate/wash samples of a 17-year-old female with CF mutations F508 and R1162X. The patient was diagnosed with CF in early childhood and had endured prolonged hospitalizations caused by severe recurrent pulmonary exacerbations and infections caused by MDR mucoid *P*. *aeruginosa* for which she had received multiple courses of antibiotic treatment. Notably, she had a history of allergies and intolerance to multiple antimicrobial agents, including ciprofloxacin, aztreonam, levofloxacin, piperacillin-tazobactam, vancomycin, and anaphylaxis to several cephalosporins. The course of her illness was marked by several CF-related complications including rapidly progressive lung disease, allergic bronchopulmonary aspergillosis (ABPA), chronic sinusitis, exocrine pancreatic insufficiency, liver disease, diabetes, and intrahepatic biliary stones necessitating multiple gastro-intestinal surgeries. During the study period, the patient received multiple antibiotic treatment regimens (Fig 1) and underwent a double-lung transplant (see S1 Appendix).

### Antimicrobial susceptibility testing

Clinical strains previously isolated by the microbiology laboratory were grown on *Pseudomonas* isolation agar (Cat No. 17208, Sigma-Aldrich, St. Louis, MO, USA). Single colonies were obtained and thereafter inoculated into cation adjusted Mueller Hinton broth and grown at 37°C overnight. Frozen bacterial stock solutions were stored at -80° C until further analysis. Automated MIC determination using MicroScan WalkAway system or Sensititre Custom Panels was performed according to Clinical and Laboratory Standards institute (CLSI 2018) methods [15]. Antimicrobial agents evaluated include piperacillin/tazobactam, ticarcillin/clavulanate, ceftazidime/avibactam, ceftolozane/tazobactam, ceftazidime, cefepime, imipenem, meropenem, aztreonam, ciprofloxacin, levofloxacin, gentamicin, tobramycin, amikacin, colistin, polymyxin B, and fosfomycin.

#### Genome sequencing

Total DNA was extracted using the MasterPure Gram Positive DNA purification kit by following manufacturer’s instructions (Epicentre, Madison, WI, USA). Libraries were prepared for sequencing using the Illumina NexteraXT kit (Illumina Inc., San Diego, CA) and 2x150 paired end reads were generated using an Illumina NextSeq550 at the Genomics Core at Case Western Reserve University. De novo assembles were performed with Velvet [16], followed by annotation with Prokka [17]. Antibiotic resistance genes were identified using the Comprehensive Antibiotic Resistance Database (CARD) [18]. Shared and variable gene content was determined using the pan-genome analysis program Roary [19].

#### Phylogenetic analysis

Among complete *P*. *aeruginosa* genome sequences, PA1RG (GenBank accession CP012679.1) was determined to be the closest relative to the 18-strain group based on BLAST analysis at NCBI. SNVs were identified by comparison of primary sequence reads to the PA1RG reference genome using the GATK HaplotypeCaller [20], SNIPPY (https://github.com/tseemann/snippy), and PARSNP [21]. SNVs with alternate alleles supported by at least two algorithms were retained. SNV sites were filtered to exclude locations that were tri-allelic, or that had reads supporting >1 allele in any individual genome. The functional impact of SNVs was assessed using snpEff [22]. The evolutionary history was inferred using the Neighbor-Joining method [23]. The percentage of replicate trees in which the associated taxa clustered together in the bootstrap test (500 replicates) are shown next to the branches. There was a total of 424 positions in the final dataset. Evolutionary analyses were conducted in MEGA7 [24]. To calculate the selection coefficient (*dN/dS* ratio) on various branches, we used the method of Nei and Gojobori [25], with the assumption that the codon usage was the same as in the reference genome *P*. *aeruginosa* PAO1 [26], where 25% of mutation sites were determined to be synonymous [27].

#### RNAseq analysis

Total RNA was extracted using Qiagen RNeasy Protect Bacteria Mini Kit and sequencing libraries were prepared using NEBNext Ultra II Directional RNA library prep kits in combination with NEBNext rRNA depletion kits specific for bacterial ribosomal RNA (New England BioLabs). RNAseq libraries were sequenced on Illumina MiSeq with 2x150 base reads. Sequence reads were quality and adapter trimmed using Trimmomatic v0.32 [28] and mapped to the Pae_AZ01 genome using bwa [29]. The number of reads per annotated coding region was determined using feature Counts v1.22.2 [30]. The isolates were grouped by hierarchical clustering of the gene expression data using the hclust algorithm in Complex Heatmap v2.10.0 [31]. Differential expression analysis was then performed using each group of isolates as the reference, compared against “all other groups” to determine variably expressed genes among groups within the EdgeR package v2.10.0 [32]. All significant genes for each group of isolates were entered into PANTHER 14.1 [33] to test for statistical overrepresentation of Gene Ontology Biological Process categories [34, 35] compared to proportions of gene annotations present in the reference PAO1 genome. Complex Heatmap was again used to show the expression of the resulting genes across isolates.

#### Ethics statements

IRB approval and informed consent were not obtained since this work did not constitute human subject research. The study did not involve interaction with human subjects nor include research on identifiable human material. No interventions or interactions with subjects occurred for the purpose of biospecimens or data collection. Clinical observation and/or surveys were not employed as part of this study. No identifiable private information or identifiable bio-specimens were obtained, used, studied, analyzed, or generated for the purpose of this study. The study involved only secondary research not collected specifically for this study and using biospecimens pertaining to a deceased individual. Specifically, all procedures and experiments were conducted in the laboratory on archived isolates. The treatment plan for infections caused by these archived frozen bacterial strains was decided by the patient’s treating physician and was not altered as a result of this study. Identifiable information was not available in the research laboratory and was therefore not included in the write-up of this manuscript. To further protect patient anonymity, all archived samples are codified with unique laboratory numbers that do not correspond to patient health information (PHI). Additionally, the decision to forego informed consent pertaining to prior archived samples serves to further protect the patient’s health information.

## Results

### Phenotypic characteristics of the recovered *P*. *aeruginosa* isolates

Twenty-two sequential *P*. *aeruginosa* isolates were obtained (pre- and post-transplant) from the patient within the 17-month observation period (Fig 1). After 18 hours of incubation at 37°C, all isolates grew abundantly on sheep blood agar, Mueller Hinton agar, and *Pseudomonas Isolation Agar*. Colonies appeared extensively mucoid in texture with fringed edges. Hemolysis was apparent on blood culture plates. Most colonies were also characterized by a “metallic sheen”, blue-green pigmentation, and a fruity odor. Uniform automated MIC testing was performed on all but one isolate, which did not grow from the frozen stock.

As summarized in Table 1, the majority of isolates were resistant to almost all tested β-lactam agents. All isolates were resistant to meropenem with MICs ≥ 8μg/ml. Uniform non-susceptibility to aztreonam, piperacillin/tazobactam, and imipenem was observed in all but two isolates. While all isolates were non-susceptible to gentamicin, ten of the 21 were found to be susceptible to tobramycin. Three isolates exhibited polymyxin B MICs ≥4 μg/ml whereas fosfomycin MICs were uniformly >64 μg/ml. Only three isolates were susceptible to ceftazidime/avibactam and ceftolozane/tazobactam (Pae_AZ01, Pae_AZ02, Pae_AZ03). These were collected during the pre-transplant period and belonged to two different clades (A and C). Interestingly, two of those three isolates were concomitantly resistant to colistin and polymyxin B (Pae_AZ02 and Pae_AZ03). Susceptibility to tobramycin varied during the study period among strains belonging to clade C; however, no specific genetic mechanism could be ascribed to this.

**Table 1:** Antimicrobial susceptibility profiles and PDC variants found in *P*. *aeruginosa* isolates.

**ID**	**Isolation date**		**Source**	**Clade**	**PDC**		**Other AMR genes**
					**Variant**	**Aminoacid substitutions (SANC)**	
Pae_AZ01	Pre-transplant	2/23/17	SPU	A	New 1	R53Q, T70I, T79A	aph(3’)-Iib, blaOXA-486, fosA, catB7
Pae_AZ02		3/14/17	SPU	Not sequenced			
Pae_AZ03		4/8/17	SPU	C	New 2	R53Q, T79A, F121L, E219K	aph(3’)-Iib, blaOXA-486, fosA, catB7
Pae_AZ04		4/8/17	SPU	C	New 3	T79A, E219K	aph(3’)-Iib, blaOXA-486, fosA, catB7
Pae_AZ05		5/27/17	SPU	C	New 2	R53Q, T79A, P153L, E219K	aph(3’)-Iib, blaOXA-486, fosA, catB7
Pae_AZ06	Post-transplant	6/7/17	B-WA	Not sequenced			
Pae_AZ07		7/7/17	BAL	C	New 2	R53Q, T79A, P153L, E219K	aph(3’)-Iib, blaOXA-486, fosA, catB7
Pae_AZ08		8/3/17	BAL	C	New 4	R53Q, T70I, T79A, P153L, E219K	aph(3’)-Iib, blaOXA-486, fosA, catB7
Pae_AZ09		8/7/17	BAL	C	New 2	R53Q, T79A, P153L, E219K	aph(3’)-Iib, blaOXA-486, fosA, catB7
Pae_AZ10		8/21/17	BAL	B	New 5	R53Q, T79A, D81N, ΔG212, E219K	aph(3’)-Iib, blaOXA-486, fosA, catB7
Pae_AZ11		8/24/17	BAL	C	New 2	R53Q, T79A, P153L, E219K	aph(3’)-Iib, blaOXA-486, fosA, catB7
Pae_AZ12		9/25/17	B-Asp	Not sequenced			
Pae_AZ13		10/15/17	Nasal	C	New 2	R53Q, T79A, P153L, E219K	aph(3’)-Iib, blaOXA-486, fosA, catB7
Pae_AZ14		11/1/17	BAL	B	New 5	R53Q, T79A, D81N, ΔG212, E219K	aph(3’)-Iib, blaOXA-486, fosA, catB7
Pae_AZ15		11/30/17	BAL	C	New 2	R53Q, T79A, P153L, E219K	aph(3’)-Iib, blaOXA-486, fosA, catB7
Pae_AZ16		12/9/17	SPU	B	New 5	R53Q, T79A, D81N, ΔG212, E219K	aph(3’)-Iib, blaOXA-486, fosA, catB7
Pae_AZ17 *		12/20/17	BAL	C	New 2	R53Q, T79A, P153L, E219K	aph(3’)-Iib, blaOXA-486, fosA, catB7
Pae_AZ18		1/5/18	SPU	C	New 2	R53Q, T79A, P153L, E219K	aph(3’)-Iib, blaOXA-486, fosA, catB7
Pae_AZ19		1/15/18	SPU	C	New 2	R53Q, T79A, P153L, E219K	aph(3’)-Iib, blaOXA-486, fosA, catB7
Pae_AZ20		1/25/18	B-WA	C	New 2	R53Q, T79A, P153L, E219K	aph(3’)-Iib, blaOXA-486, fosA, catB7
Pae_AZ21		2/21/18	SPU	C	New 2	R53Q, T79A, P153L, E219K	aph(3’)-Iib, blaOXA-486, fosA, catB7
Pae_AZ22		3/4/18	B-WA	C	New 2	R53Q, T79A, P153L, E219K	aph(3’)-Iib, blaOXA-486, fosA, catB7

**ID**	**Minimum inhibitory concentrations (MICs, mg/L)**																	**Classification**
	**Piperacillin-tazobactam**	**Ticarcillin/ Clavulanate**	**Ceftazidime/ Avibactam**	**Ceftolozane/ Tazobactam**	**Cefepime**	**Ceftazidime**	**Imipenem**	**Meropenem**	**Aztreonam**	**Ciprofloxacin**	**Levofloxacin**	**Gentamicin**	**Tobramycin**	**Amikacin**	**Colistin**	**Polymyxin**	Fosfomycin**	
Pae_AZ01	32	**>128**	≤2	≤2	**>16**	**>32**	**>8**	**16**	**>32**	**>2**	**>8**	**>8**	8	32	2	2	**>64**	XDR
Pae_AZ02	16	64	≤2	≤2	8	4	**>8**	**8**	16	0.5	2	8	≤1	16	**>4**	**>4**	**>64**	MDR
Pae_AZ03	16	**128**	4	≤2	16	16	**>8**	**8**	16	0.5	2	8	≤1	16	**>4**	**>4**	**>64**	MDR
Pae_AZ04	**>64**	**>128**	**>16**	**>8**	**>16**	**>32**	**>8**	**>32**	**>32**	**>2**	**>8**	**>8**	4	**>32**	2	1	**>64**	MDR
Pae_AZ05	32	**>128**	**>16**	**>8**	**>16**	**>32**	4	**8**	**>32**	**>2**	**>8**	**>8**	2	**>32**	0.5	1	**>64**	MDR
Pae_AZ06	**>64**	**>128**	**>16**	**>8**	**>16**	**>32**	**8**	**16**	**>32**	**>2**	**>8**	**>8**	4	**>32**	2	1	**>64**	MDR
Pae_AZ07	64	**>128**	**>16**	**>8**	**>16**	**>32**	≤1	**16**	**>32**	**>2**	**>8**	**>8**	2	**>32**	2	1	**>64**	MDR
Pae_AZ08	**>64**	**>128**	**>16**	**>8**	**>16**	**>32**	**8**	**8**	**>32**	**>2**	**>8**	**>8**	4	**>32**	2	4	**>64**	MDR
Pae_AZ09	**>64**	**>128**	**>16**	**>8**	**>16**	**>32**	**>8**	**>32**	**>32**	**>2**	**>8**	**>8**	4	**>32**	2	1	**>64**	MDR
Pae_AZ10	**>64**	**>128**	**>16**	**>8**	**>16**	**>32**	**8**	**>32**	**>32**	**>2**	**8**	**>8**	**>8**	**>32**	1	1	**>64**	MDR
Pae_AZ11	**>64**	**>128**	**>16**	**>8**	**>16**	**>32**	2	**32**	**>32**	**>2**	**>8**	**>8**	4	**>32**	2	1	**>64**	MDR
Pae_AZ12	**>64**	**>128**	**>16**	**>8**	**>16**	**>32**	**8**	**32**	**>32**	**2**	**>8**	**>8**	**>8**	**>32**	0.5	1	**>64**	MDR
Pae_AZ13	**>64**	**>128**	**>16**	**>8**	**>16**	**>32**	**8**	**32**	**>32**	**>2**	**>8**	**>8**	4	**>32**	2	2	**>64**	MDR
Pae_AZ14	**>64**	**>128**	**>16**	**>8**	**>16**	**>32**	**8**	**>32**	**>32**	**2**	**4**	**>8**	**>8**	**>32**	1	1	**>64**	MDR
Pae_AZ15	**>64**	**>128**	**>16**	**>8**	**>16**	**>32**	4	**32**	**>32**	**>2**	**>8**	**>8**	**>8**	**>32**	2	1	**>64**	MDR
Pae_AZ16	**>64**	**>128**	**>16**	**>8**	**>16**	**>32**	**8**	**>32**	**>32**	**>2**	**8**	**>8**	**>8**	**>32**	1	1	**>64**	MDR
Pae_AZ17 *	--	--	--	--	--	--	--	--	--	--	--	--	--	--	--	--	--	--
Pae_AZ18	**>64**	**>128**	**>16**	**>8**	**>16**	**>32**	**>8**	**>32**	**>32**	**>2**	**>8**	**>8**	**>8**	**>32**	2	1	**>64**	MDR
Pae_AZ19	32	**>128**	**>16**	**>8**	**>16**	**>32**	**>8**	**>32**	**>32**	**>2**	**>8**	**>8**	**>8**	**>32**	1	1	**>64**	MDR
Pae_AZ20	**>64**	**>128**	**>16**	**>8**	**>16**	**>32**	**>8**	**>32**	**>32**	**>2**	**>8**	**>8**	**>8**	**>32**	2	2	**>64**	MDR
Pae_AZ21	**>64**	**>128**	**>16**	**>8**	**>16**	**>32**	**>8**	**32**	**>32**	**>2**	**>8**	**>8**	**>8**	**>32**	1	1	**>64**	MDR
Pae_AZ22	**>64**	**>128**	**>16**	**>8**	**>16**	**>32**	**>8**	**>32**	**>32**	**>2**	**>8**	**>8**	**>8**	**>32**	2	2	**>64**	MDR

SPU: sputum; B-WA, bronchial wash; BAL, bronchoalveolar lavage; B-Asp, bronchial aspirate.

* Isolate could not be regrown from frozen stock. NS, not sequenced.

Numbers in bold indicate resistant according to CLSI breakpoints, except for fosfomycin (** Ecoff value as per EUCAST).

#### Genomics and transcriptomics

**Fig 2:**
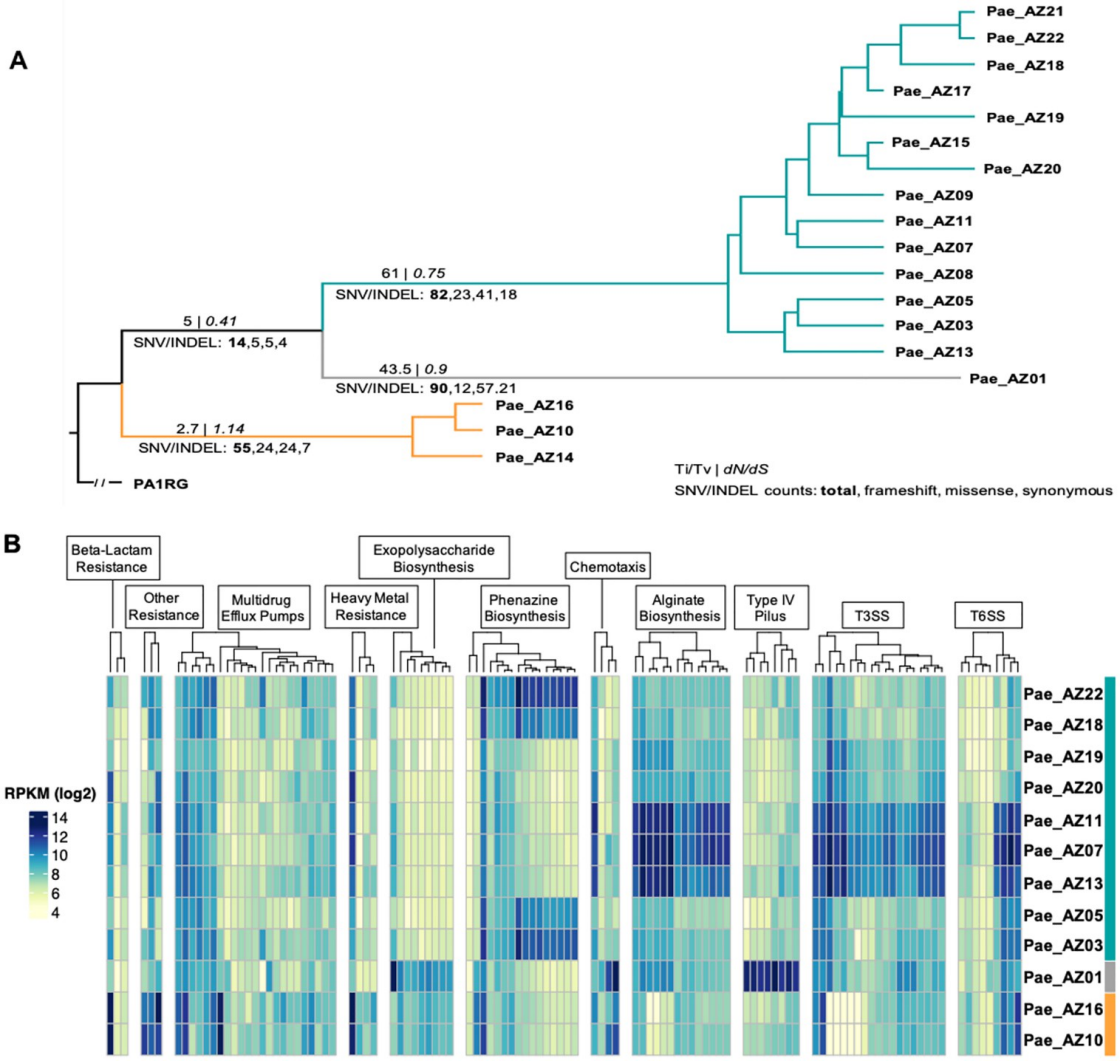
**A. Neighbor-Joining tree indicating relatedness among sequenced isolates**. Tree was built in MEGA using a concordant set of 424 SNVs from GATK, Snippy, and Parsnp with PA1RG as the reference sequence. Three main clades shown are Clade A (gray), Clade B (orange), and Clade C (teal). On each branch, the transition to transversion ratio (Ti/Tv) and the *dN/dS* ratio are shown above the line, with total numbers of variants of each type below the line. The percentage of replicate trees in which the associated taxa clustered together in the bootstrap test (500 replicates) are shown next to the branches. **B. Expression patterns of antibiotic resistance/pathogenicity genes amongst *P*. *aeruginosa* isolates**. RNA-seq data from 12 isolates representing all 3 clades showed variable gene expression in multiple resistance and pathogenicity functional categories (indicated at top). Hierarchical clustering of the gene expression data was used to group isolates for differential expression (DE) analysis. Differentially expressed genes were tested for overrepresentation of Gene Ontology Biological Process categories compared to the PAO1 genome in PANTHER; DE and overrepresented categories are shown.

Nineteen isolates were further analyzed by whole genome sequencing, and 12 of those were also analyzed by RNAseq. One isolate (Pae_AZ04) obtained before transplantation, differed from the remaining genomes at more than 30,000 sites, indicating that it represented a different strain. A phylogenetic tree was constructed for the remaining 18 genomes using 424 phylogenetically informative variants (S2 Table) that revealed three clades with 1, 3, and 14 members that share a recent common ancestor (clades A, B, and C, respectively; Fig 2A). Genes conferring resistance to antibiotics could be identified using CARD. All collected isolates were found to harbor aph(3’)-XV and aph(3’)-II which encode the protein aminoglycoside 3’-phosphotransferase; an aminoglycoside modifying enzyme (AME). Genes encoding porin channel proteins modulating permeability and/or membrane efflux pump were also generated through CARD and were homogenously present in all isolates. These are not explicitly listed since resistance mediated via efflux and/or porin channel mechanisms depends on regulatory processes conferring various expression levels. Notably, a general trend toward accumulation of mutations with time was observed. Two clades (A and C) uniquely manifested different mutations in the DNA mismatch repair gene *mutL* and exhibited an elevated ratio of transition to transversion mutations which comprise hallmark features of the hypermutator phenotype [36–38] (Table 2).

**Table 2:** Evolutionary parameters of *P*. *aeruginosa* genomes.

Clade	Ti/Tv^1^	%indel^2^	in-frame indel	frameshift	missense	synonymous	intergenic	*dN/dS*
**Pae_AZ01 (A)**	43.5	19	3	12	57	21	17	0.90
**Pae_AZ14/ Pae_AZ16/ Pae_AZ10 (B) *mutL*-WT**	2.7	52	11	24	24	7	4	1.14
**14-strain group with *mutL*-fs (C)**	37.3	26	4	35	78	30	19	0.87
**Shared by clades A+C**	4.6	15	7	6	23	45	5	0.17
**Pae_AZ04**	2.0	2	74	167	4703	16384	3452	0.04
**PA1RG**	3.5	7.8	15	35	3864	13368	2855	0.09

^1^Ratio of transition mutations to transversion mutations among all SNVs;

^2^Percentage of insertion/deletion mutation.

The overall *dN/dS* ratio, an indication of the strength of natural selection, was >1 only in clade B, indicative of strong positive selection for novel mutations in these isolates (Table 2). The two clades with *mutL* mutations (A and C) have increased *dN/dS* ratios relative to the background *dN/dS* rate of variants shared by these genomes, compared to the closest unrelated complete reference genome PA1RG.

**Fig 3:**
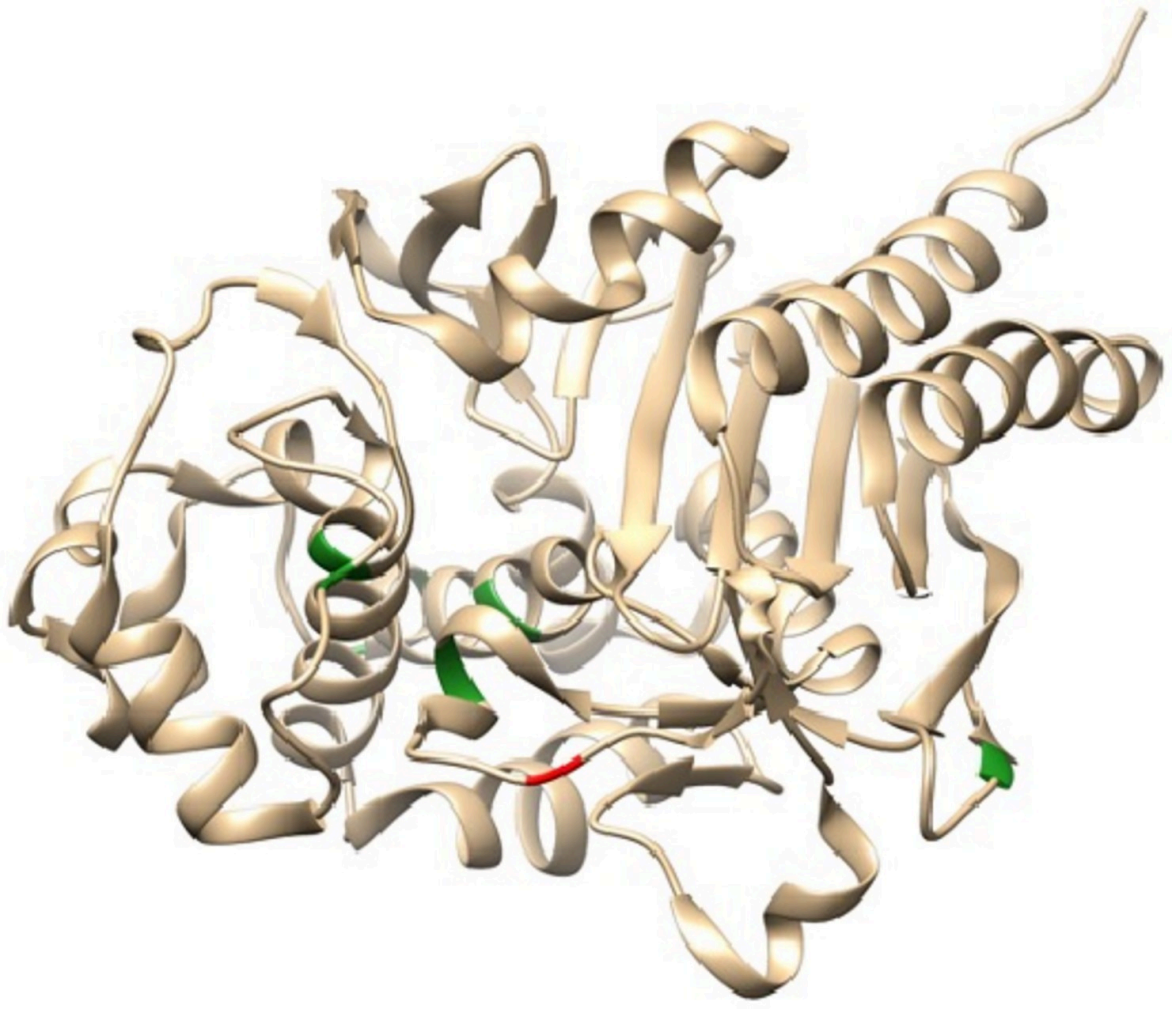
PDC structure (PDB ID 4GZB) showing the region where the deletion (red) and substitutions listed in Table 1 (green) where found.

Interestingly, the *ampC* β -lactamase gene (*bla*_PDC_) was the most changed overall with five different nonsynonymous mutations and one deletion identified and no synonymous changes, resulting in 5 new variants with 2–5 amino acid substitutions as compared to PDC-1 (S2 Table, Fig 3). Other genes involved in antibiotic resistance with multiple nonsynonymous mutations were *gyrA* (3), *mexB* (5), and *mexY* (4).

Transcriptome profiling by RNAseq performed on representative isolates from each clade, revealed substantial differences in the expression of genes associated with antibiotic resistance and virulence traits in various isolate subsets (Fig 2B, S3 Table). Clade C isolates demonstrated notable variable expression of several virulence-related genes. For example, Pae_AZ11, Pae_AZ07, and Pae_AZ13 exhibited increased expression of the Type IV pilus, Type III secretion system and Type VI secretion system, which encodes toxin effector VgrG genes. Increased expression of alginate biosynthesis genes was seen in isolates Pae_AZ22, Pae_AZ18, Pae_AZ05, and Pae_AZ03,however, this did not morphologically correlate with a more mucoid appearance on agar plates. The expression of phenazine and exopolysaccharide biosynthesis genes was elevated in the isolate Pae_AZ01. Analysis of *bla*_PDC_ gene expression identified significant variability (up to 50-fold) across all isolates. Clade B demonstrated the highest *bla*_PDC_ expression, while several other isolates exhibited 2-10x higher expression compared to Pae_AZ01.

## Discussion

Whole genome sequencing and RNAseq were successfully employed to characterize the underlying genomic population diversity and define the nature of sequence changes over time in a series of *P*. *aeruginosa* isolates obtained from a CF patient over a 17-month period (Fig 1A).The diversity of mutated genes and pattern of changing antibiotic resistance phenotypes, implied functional divergence among the isolates. Transcriptome profiling by RNAseq (Fig 2B) revealed significant differential expression in genes associated with several antibiotic resistance and virulence traits in various isolate subsets and comprehensively outlined the impact of the accumulated mutational load. Notably, increased expression of alginate exopolysaccharide and phenazine biosynthesis was observed. The role of alginate production on conversion to mucoid *P*. *aeruginosa* phenotypes, tissue damage, biofilm growth, and decreased clearance by host immune response has been well studied. in the pathogenesis of *P*. *aeruginosa* has been well studied. The alginate-containing biofilm has been shown to protect *P*. *aeruginosa* from phagocytosis and impair antibiotic penetration thereby enabling persistence in airways and chronic lung infection [39, 40] In addition, *P*. *aeruginosa* in biofilm-based infections releases phenazines, metabolites that accept electrons to support cellular redox balancing which allows for metabolic heterogeneity. This in turn, promotes tolerance to antibiotics causing differential antibiotic susceptibilities which further impede the treatment of biofilm-based infections [41].

Most collected isolates met the more recently described definitions/criteria for DTR *P*. *aeruginosa*. The patient received multiple courses of antimicrobial therapy, often administered as combinations of two or more agents. Therefore, delineating specific associations between individual antimicrobial agents and the accumulation of mutations within sequenced genomes was challenging. Similarly, it was not possible to definitively link specific mutations with either prevailing or incipient resistance phenotypes. Phylogenetic tree construction for 18 genomes revealed three clades representing a genotypically heterogenous population as illustrated in Fig 2A. The *mutL* wild-type strains within clade B exhibited higher expression of *ampC* (*bla*_PDC_), several efflux pump genes, and exopolysaccharide biosynthesis *psl* operon compared to Clade C strains. Conversely, analysis of clade B genomes showed lower or absent expression of genes related to the Type IV pilus and Type III secretion system.

Overall, the pattern of mutation accumulation and variation of gene expression suggests that a progenitor strain was present in the patient prior to transplantation. The analysis demonstrates continued evolution of this strain in response to multiple pressures throughout the course of treatment. Rather than following a strictly orderly progression of increased resistance and/or virulence, the gene expression patterns indicated considerable functional specialization that appears to have been driven by diversifying selection based on elevated *dN/dS* ratios, including in the group of strains without a *mutL* mutation. There is growing evidence that *P*. *aeruginosa* populations in the lungs of CF patients exhibit high levels of phenotypic diversity and that divergent sublineages can coexist within the spatially structured and heterogeneous lung environment [42, 43]. Diversification in the context of infection was described by Jorth, *et al*. [44], who ascribed the differences to regional isolation of bacterial sub-populations that experienced varying selection based on nutrient availability, host defense and antibiotic resistance, as well as virulence linked to the type III secretion system. Other studies have found that different *P*. *aeruginosa* lineages undergo convergent molecular evolution in a group of genes that suggests a role in host adaptation for remodeling of regulatory networks and central metabolism, acquisition of antibiotic resistance, and loss of extracellular virulence factors [45].

Our analyses highlight the need for thorough initial sampling of population heterogeneity when gathering microbiological data. The underlying heterogeneity demonstrated via phylogenetic reconstruction indicates that the profiling of a single isolate to inform antibiotic therapy may potentially yield ineffective treatment against other unsampled populations, which hastens further resistance [42]. While it is still not yet clear how many samples and which mode of collection best captures this heterogeneity, a single sample is almost certainly too few [46].

**Fig 4:**
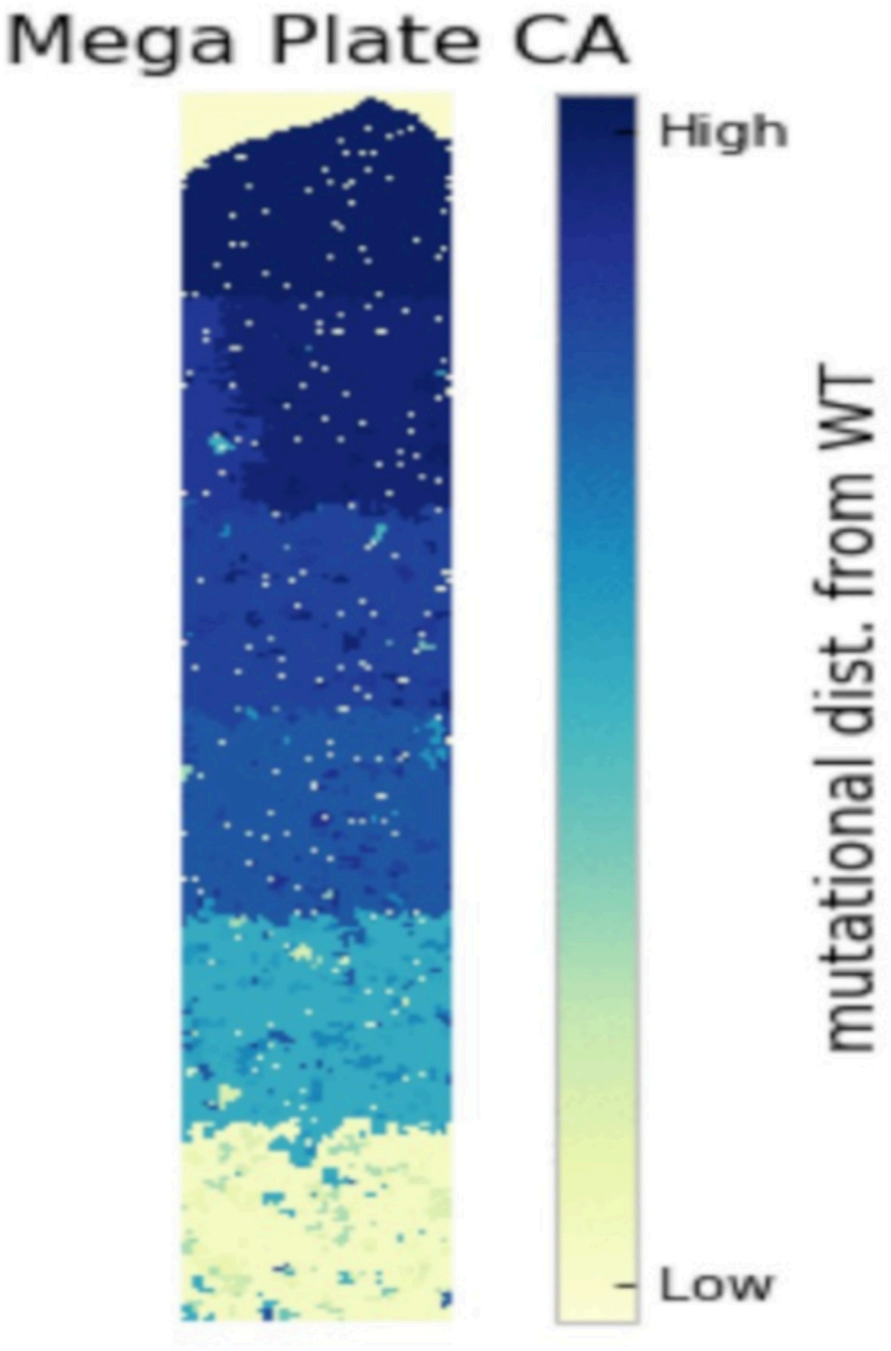
Spatiotemporal environmental heterogeneity can drive genetic load and ultimately evolution of drug resistance in a bacterial population. A snapshot of a cellular automata model of evolution in which individuals are capable of rare mutations that confer resistance to antibiotics, as they begin in a low antibiotic concentration region and spread through discrete patches of increasing concentration. Increasing antibiotic concentration drives the fixation of resistance mutations which allow individuals on the frontier of the colonizing population to survive when confronted with higher antibiotic concentration than they are currently able to tolerate.

To address short evolutionary timescales, emerging rapid antimicrobial susceptibility testing, whole genome sequencing methods, and RNAseq are important strategies to close the time lag between microbiological sampling and treatment [47–50]. We further postulate that more robust assays (phenotypic and genotypic) would determine the probability distribution of *future* evolved phenotypes rather than current phenotype to facilitate selection of the most appropriate antibiotic given the set of evolutionary possibilities [51, 52]. For example, evolution is inherently stochastic and thus prior chance events (i.e., mutations) can influence future evolutionary outcomes in sometimes unpredictable ways [53]. Recent work has highlighted this process of historical contingency by using experimental populations of *Escherichia coli* that independently evolved for several decades in an environment without antibiotics [54, 55]. These strains had typically become more susceptible to several different antibiotics relative to their common ancestor during this period of relaxed selection. Moreover, evolvability was idiosyncratic with respect to initial genotype, such that resistance was more constrained in some backgrounds than in others [54]. Background also affected the genomic basis of resistance. On average, replicate lines evolved from the same founding genotypes had more mutations in common at the gene level than did lines evolved from different founding genotypes [55]. A lineage’s evolutionary history can therefore influence both its phenotypic and genotypic paths to antibiotic resistance (Fig 4). In addition, previous theoretical work has demonstrated that with complete knowledge of possible drug resistance profiles conferred by particular genotypes, drug sequences can be designed to systematically steer evolution away from resistance [56, 57]. Could these strategies be applied to patients? Aside from drug susceptibility and genome sequencing, other data types may also be informative to this end. As the rate of change of mRNA expression, or RNA velocity, has been used to characterize terminal cell fates in experimental models, pathogen RNA expression profiles may provide clinically important data on future antibiotic susceptibility [57].

Our findings reveal unexpected complexity, where phenotypes change in an unpredictable manner unrelated to antibiotic treatment, thereby challenging antimicrobial stewardship programs that assist with the selection and duration of antibiotic regimens in critically ill and immunocompromised patients based on limited laboratory-derived resistant profiles. We submit that an approach sampling the population of pathogens present in a clinical sample be applied when dealing with DTR- *P*. *aeruginosa* [58–61]. Since there was little difference in antibiotic susceptibility across isolates, the genome/transcriptome data provided a much richer view of the extent of heterogeneity among isolates. This highlights the potential value of timely inclusion of molecular characterization in a clinical setting. The complexity of choosing appropriate antibiotics with imprecise data based on single-isolate susceptibility testing, even despite efforts to unravel the mechanism, shows that novel approaches are needed. In complex infections such as the one presented by this young woman with CF, a combination of real-time testing and genomic/transcriptomic will lead in the near future to the application of true “precision medicine” by helping clinicians choose the combination antimicrobial therapy most likely to be successful against a population of MDR pathogens.

## Supporting information

S1 Table Assembly statistics. (XLSX)

S2 Table SNPs and INDELs. (XLSX)

S3 Table RPKM values for selected genes. (XLSX)

S1 Appendix Clinical case. (DOCX)
