# Supplementary material for: Genomic heterogeneity underlies multidrug resistance in Pseudomonas aeruginosa: A population-level analysis beyond susceptibility testing
Source: PLoS One. 2022 Mar 31;17(3):e0265129. doi: 10.1371/journal.pone.0265129 (PMC8970513; doi:10.1371/journal.pone.0265129)
Supplement: S1 Appendix — (DOCX) [file pone.0265129.s004.docx]

**Supplementary appendix**

**CLINICAL CASE**

Pre-transplant

The patient was a 17-year-old woman with cystic fibrosis (CF), (heterozygous for CFTR mutations F508del and R1162X) diagnosed in early childhood, who presented with a severe pulmonary exacerbation with increased sputum production and weight loss requiring hospitalization. She had a history significant for rapidly progressive lung disease, allergic bronchopulmonary aspergillosis (ABPA), chronic sinusitis, exocrine pancreatic insufficiency, CF-related liver disease, CF-related diabetes, and intrahepatic biliary stones with more than a dozen gastro-intestinal (GI) surgeries leaving her with multiple intra-abdominal adhesions. She had a history of allergies and intolerances to multiple antimicrobial agents, including reactions to ciprofloxacin, aztreonam, levofloxacin, piperacillin-tazobactam, vancomycin, and anaphylaxis to several cephalosporins. Her lungs were chronically infected with MDR mucoid *P. aeruginosa* for which she received multiple courses of antibiotic treatment in her native country Venezuela. For the last 2 years, she was also being followed at a CF center in the US every 3-6 months, co-managing her disease with the Venezuelan providers.

Upon admission to the hospital, spirometry revealed percent predicted forced expiratory volume in 1 second (ppFEV1) of 47 % (a drop from 72 %), and she required 4L/min of O_2_ via nasal cannula (NC) for hypoxia. She had failed 4 courses of 14-21 days (total of 6 months) of intravenous (IV) and inhaled antibiotic therapy including tobramycin (IV and inhaled), ceftazidime (IV), colistin (IV and inhaled), aztreonam (IV and inhaled), meropenem (IV), cefepime (IV), and levofloxacin (IV) in rapid sequence as well as a trial of systemic steroids for presumed ABPA. Her sputum culture was positive for *P. aeruginosa* (Table 1). The antimicrobial stewardship team (ASP) was consulted, and due to prior failure to all the susceptible agents with the exception of ceftolozane-tazobactam, it was determined this agent should be the backbone of her therapy. She underwent desensitization to ceftolozane-tazobactam after a positive allergen skin test to this agent. She received 11 days of ceftolozane-tazobactam, 3g IV every 8 hours (q8h), in combination with tobramycin 500mg IV (10mg/kg) q24h while admitted, with an additional 5 days as an outpatient. She responded well to this treatment, was weaned off of oxygen, and remained as an outpatient for 31 days. During this time she was managed with inhaled tobramycin and aztreonam.

Her next admission to the hospital was due to fever, vomiting, diarrhea, and fatigue, but she was also noted to have increased cough and greenish sputum, and was hypoxic requiring 3L of O_2_ via NC. Repeat cultures revealed an extremely drug resistant (XDR) *P. aeruginosa*, now resistant to all agents except for colistin, polymyxin B, ceftolozane-tazobactam, and ceftazidime-avibactam. After 4 days of oral levofloxacin 750mg q24h, she once again underwent ceftolozane-tazobactam desensitization upon return of her culture susceptibilities. She completed another 17-day course of IV ceftolozane-tazobactam and tobramycin with good clinical response. Against the advice of ASP, she was discharged on IV ceftolozane-tazobactam monotherapy for an additional 28 days, and 4 days after completion she presented again with a pulmonary exacerbation, significantly more severe than the previous two admissions.

Upon the 3^rd^ admission, spirometry was significant for a drop in ppFEV_1_ to 21% (lowest ever). Despite resuming IV ceftolozane-tazobactam and tobramycin, her clinical status continued to deteriorate as evidenced by increased oxygen requirement (2-3L O_2_ via NC), increased sputum production, pleuritic chest pain, and persistent nausea (attributed to gastroparesis). Repeat sputum cultures again grew *P. aeruginosa*, now XDR against all available agents, including ceftolozane-tazobactam and ceftazidime-avibactam with minimum inhibitory concentrations (MICs) >256µg/mL.

A multi-disciplinary meeting was held due to her rapidly worsening clinical status and progressively resistant *P. aeruginosa* strains. At this time, even with severely poor nutritional status, she was too sick from a pulmonary and GI standpoint for any surgeries, including feeding tube placement. She was deemed ineligible for lung transplantation, despite transplantation likely being her only chance of survival. Strategies such as palliative care and end of life care were addressed, as well as the importance of tackling the XDR *P. aeruginosa*. The patient’s antimicrobial regimen was altered, and the off-label use of high-dose oral fosfomycin (3g q6h) to achieve systemic effects against XDR *P. aeruginosa* was started. Institutional Review Board (IRB) approval for the use of IV fosfomycin was sought and a compassionate use Investigational New Drug Application (IND) to the FDA was submitted to obtain the agent from the United Kingdom as the intravenous formulation of fosfomycin was not FDA approved for use in the US. She was started on off-label fosfomycin, 3g orally (PO) q6h while in vitro testing was set up, understanding the limitations without breakpoints provided by CLSI or EUCAST guidelines for *P. aeruginosa*. After desensitization, ceftazidime-avibactam 2.5g IV q8h was added as combination therapy to help prevent the development of resistance to fosfomycin and aid in the treatment of the XDR *P. aeruginosa*. After 8 days of oral fosfomycin, the patient developed GI intolerance with persistent nausea and diarrhea, and treatment frequency was decreased to q8h. While easier for the patient to tolerate, there was concern regarding inadequate exposure of fosfomycin against the *P. aeruginosa* with reduced dosing.

Oral fosfomycin and ceftazidime-avibactam were continued with the addition of IV aztreonam 2g q6h while awaiting IV fosfomycin approval. She progressively improved on ceftazidime-avibactam and oral fosfomycin, and weaned off supplemental oxygen after 3 days of this therapy. Fosfomycin was transitioned to the IV formulation after IND approval on day 30 of oral fosfomycin therapy. She was placed on a dose of 4g IV q8h and continued on her other antimicrobial agents. After the addition of IV fosfomycin the patient significantly improved, demonstrating her best ppFEV_1_ in more than a year, and was able to tolerate gastro-jejunum (GJ) tube placement to improve her nutritional status.

At this point, it was determined that the best option for her future was a double lung transplant; she met the criteria to be listed, and after 77 days of hospitalization she was discharged home on IV aztreonam, IV ceftazidime-avibactam and oral fosfomycin (3g q8h). She tolerated this regimen at home for 13 days, but was re-admitted to the hospital with a recurrence of diarrhea, decreased appetite, shortness of breath on exertion, and worsening cough and sputum production. Malabsorption of oral fosfomycin was suspected, and IV fosfomycin was resumed in conjunction with aztreonam and ceftazidime-avibactam. Donor lungs became available 6 days later, and she underwent a double lung-transplant (within 2 weeks of listing and 202 days after ASP was initially consulted).

Post-transplant

The patient’s post-transplant course was complicated with posterior reversible encephalopathy syndrome (PRES) with seizures, altered mental status, and temporary loss of vision and memory. Due to these complications she could not be promptly extubated and required a tracheostomy. Her initial respiratory cultures post-transplant were negative for *P. aeruginosa,* but three weeks after transplantation, despite no signs or symptoms of infection, the tracheal aspirates and routine post-transplant surveillance bronchoalveolar lavage (BAL) revealed XDR mucoid *P. aeruginosa*. It was suspected that the source was from her upper airways, most likely her sinuses. Routine respiratory surveillance cultures were performed every two weeks and were sent for mechanism based susceptibility testing (MBST) using double disc diffusion (DDD) and triple disc diffusion (TDD) with different antibiotic combinations to help guide the next antibiotic regimen, should she require treatment. Eighty days post-transplant, she became tachypneic on exertion with associated hypoxia. A BAL culture grew *P. aeruginosa*. She was treated with antimicrobials per Figure 1 with good clinical response, and was discharged home 117 days after transplantation on her prophylactic post-transplant antimicrobials and inhaled tobramycin (figure 1).

She was readmitted 16 days after discharge for severe headache with nausea, vomiting, and blurry vision. She was treated with IV levofloxacin for 3 days without improvement. A sinus culture grew XDR *P. aeruginosa*, and she was switched to fosfomycin 3g orally q8h, and tobramycin 330mg (7mg/kg/dose) IV q24 for 7 days. She improved and was discharged to home on inhaled tobramycin. Eight days later she was readmitted with fever and a productive cough. BAL grew XDR *P. aeruginosa*, and she was treated with IV ceftazidime-avibactam, IV aztreonam, and IV and inhaled tobramycin. Inhaled colistin was added but not tolerated due to nausea and emesis and was discontinued. She improved and was discharged after 24 days of this antimicrobial regimen,

Her final re-admission occurred 4 days later. She presented with dyspnea, productive cough with green sputum, and required 3L O_2_ via NC. Chest CT showed worsening “tree-in-bud” and consolidations in both lower lobes. IV aztreonam, IV ceftazidime-avibactam, and IV and inhaled tobramycin was resumed. Bronchoscopy with BAL revealed thick purulent secretions and XDR *P. aeruginosa* (all acid-fast bacterial and fungal cultures were negative post-transplantation). Due to worsening symptoms and BAL findings, ceftazidime-avibactam was discontinued, the patient was desensitized to meropenem, and meropenem-vaborbactam, 4g IV q8h was added. Her condition worsened requiring 8L O_2_ via NC, Based on MBST results, IV tobramycin was changed to IV colistin, and all other antibiotics remained the same. With additional MBST, IV aztreonam was changed to oral fosfomycin, 3g q8h four days later, awaiting a new IND approval for IV fosfomycin. During this time, the patient again demonstrated intolerance to oral fosfomycin, developing diarrhea 6-7 times daily and decompensating from a respiratory standpoint, likely due to inadequate fosfomycin exposure. After 5 days of oral fosfomycin therapy, the patient was able to transition to IV fosfomycin 8g q8h. She significantly improved on this regimen (IV fosfomycin IV colistin, IV meropenem-vaborbactam, and inhaled tobramycin) and weaned off supplemental oxygen. Therapy was continued for 25 days. Despite 38 days total of therapy with meropenem-vaborbactam, she developed costochondritis with hypoxemia, once again requiring oxygen supplementation.

Her respiratory status and clinical picture waxed and waned during this 3-month admission. Care was eventually transitioned to comfort measures due to continuing decline with multiple complications, including choledocholithiasis, rejection of her transplant, small bowel obstruction, and hypercapnic respiratory acidosis. Unfortunately, due to these multiple complications the patient died 9 months post transplantation, despite tailored management of her XDR and PDR *P. aeruginosa*.
